# Unraveling the Easix Score: Its Association with Vasopressor Need in Critically Ill Septic Pediatric Hematology–Oncology Patients

**DOI:** 10.3390/jcm14197105

**Published:** 2025-10-09

**Authors:** Lama Elbahlawan, Majd Khiami, Chad Blackshear, Saad Ghafoor, Alexandra Schaller, Sherry Johnson, Gabriela Maron, Raktima Datta, Amr Qudeimat, Jennifer McArthur

**Affiliations:** 1 Division of Critical Care Medicine, St. Jude Children’s Research Hospital, Memphis, TN 38105, USAsaad.ghafoor@stjude.org (S.G.); jennifer.mcarthur@stjude.org (J.M.); 2 Department of Biostatistics, St. Jude Children’s Research Hospital, Memphis, TN 38105, USA; chad.blackshear@stjude.org; 3 Department of Critical Care Medicine, Lebonheur Children’s Hospital, Memphis, TN 38105, USA; 4 Department of Nursing, St. Jude Children’s Research Hospital, Memphis, TN 38105, USA; 5 Department of Infectious Disease, St. Jude Children’s Research Hospital, Memphis, TN 38105, USA; 6 Department of Bone Marrow Transplantation and Cellular Therapy, St. Jude Children’s Research Hospital, Memphis, TN 38105, USA

**Keywords:** pediatric hematology oncology patients, sepsis, EASIX score, modified EASIX score, vasopressor support, survival, intensive care unit, renal replacement therapy

## Abstract

**Background**: Sepsis is a common and serious complication in pediatric hematology oncology (PHO) patients. The Endothelial Activation and Stress Index (EASIX) score offers a potentially accessible tool for risk stratification in septic patients. Our objective was to evaluate the association of the EASIX score with adverse clinical outcomes among septic PHO patients. **Methods**: A retrospective review was conducted for all PHO patients admitted to the intensive care unit (ICU) with sepsis from July 2022 to December 2024. **Results**: A total of 53 patients with 65 sepsis events were included in this analysis. The median age was 14.9 [IQR 9.9] y and the most common disease was hematologic malignancy. In our cohort, 60% needed vasopressors, 36% required invasive mechanical ventilation, and 22% underwent renal replacement therapy (RRT). Log2-EASIX > 2.5 was associated with higher vasopressor requirements (87% versus 45% in the low log2-EASIX group, *p* = 0.001) and an increased need for RRT (39% versus 12%, *p* = 0.024). Septic PHO patients with log2-EASIX > 2.5 were 6.9 times more likely to require vasopressor support [(95% CI 1.7–27.8) *p* = 0.007]. In addition, PHO patients with log2-EASIX > 2.5 had a longer ICU stay (7 d versus 2 d in the low log2-EASIX group, *p* = 0.024) and an extended overall hospitalization (33 d versus 25 d, *p* = 0.029). **Conclusions**: A higher EASIX score was associated with adverse outcomes in critically ill septic PHO patients. Our findings suggest that the EASIX score can be used as a tool for identifying septic patients at an increased risk of clinical deterioration and poor outcomes. Prospective studies in larger cohorts are warranted to validate and expand upon these findings.

## 1. Introduction

Sepsis is a common complication in pediatric hematology oncology (PHO) patients, with particularly high mortality rates of 41–46% when they require ICU admission [1]. These patients are especially vulnerable to infections due to their immunocompromised status and require immediate evaluation and treatment when sepsis is suspected. A rapid recognition and reversal of shock are crucial to reduce the risk of multiorgan dysfunction and death. In addition, these patients are at an increased risk of endothelial dysfunction. At the molecular level, endothelial cells in pediatric oncology patients, particularly during hematopoietic cell transplantation (HCT), suffer injury from the cytotoxic effects of chemotherapy agents, such as alkylating agents and anthracyclines, as well as from total-body irradiation. These therapies produce reactive oxygen species, cause DNA strand breaks, and damage the endothelial glycocalyx [2,3,4]. The loss of the glycocalyx layer exposes subendothelial collagen and tissue factors, which drive the activation of the coagulation cascade through ultra-large von Willebrand factor-mediated platelet adhesion [2,4]. Angiopoietin-1 (Ang1) serves as the primary agonist for the endothelial Tie-2 receptor, facilitating its autophosphorylation to reinforce intercellular junctions, boost basement membrane interactions, and promote vascular stability. Conversely, angiopoietin-2 (Ang2) competes for Tie-2 binding but does not fully activate downstream signaling pathways, thereby undermining cytoskeletal support and junctional integrity. An elevated Ang2:Ang1 ratio diminishes Tie-2 activity, disrupts vascular endothelial cadherin-based adherens junctions, increases endothelial permeability, and facilitates leukocyte extravasation. This imbalance in the angiopoietin axis is crucial to barrier dysfunction in conditions such as sepsis, graft-versus-host disease, and transplant-associated microangiopathies [5,6]. Furthermore, concurrent complement activation, which leads to the formation of a membrane attack complex, exacerbates endothelial injury and maintains a proinflammatory, prothrombotic environment [7]. In hepatic sinusoids, these pathological processes result in sinusoidal obstruction syndrome (SOS) [4], while, in cardiac, renal, pulmonary, and cerebral microvasculature, they contribute to transplant-associated thrombotic microangiopathy, leading to organ toxicity [7]. Clinically, such endothelial dysfunction manifests as fluid overload, refractory hypotension, and multi-organ failure.

Composite indices like the Endothelial Activation and Stress Index (EASIX) provide a quantifiable measurement of overall endothelial distress by integrating markers of cellular injury, renal endothelial strain, and platelet consumption. Initially validated in recipients of allogeneic HCT, elevated EASIX values have been shown to reliably predict SOS, non-relapse mortality, and severe graft-versus-host disease, thereby allowing for preemptive interventions—such as the use of defibrotide or complement inhibitors—to prevent irreversible organ damage [8,9]. The pathophysiological similarities observed in sepsis further support the prognostic relevance of EASIX beyond the transplant context. In septic conditions, pathogen-associated molecular patterns activate endothelial Toll-like receptors, triggering a cascade of cytokine release (including TNF-α and IL-1β) that accelerates glycocalyx shedding, compromises intercellular junctions, and promotes microvascular thrombosis and capillary leakage [10]. By reflecting these disturbances through markers of cellular injury (such as LDH), renal endothelial impairment (creatinine levels), and platelet consumption, EASIX has been shown to correlate independently with both 28-day and long-term mortality in critically ill septic patients, highlighting its utility for risk stratification and therapeutic decision-making across various states of endothelial stress [11].

The current sepsis guidelines emphasize the urgency of early interventions, including fluid resuscitation and the timely initiation of antimicrobial therapy [12]. Additionally, in a recently published international consensus statement, a panel of experts highlighted the importance of early recognition and prompt ICU transfer for HCT and immune effector cell therapy patients (HCT_IEC) [13]. Screening tools—such as vital sign-based alerts and clinical assessments—are increasingly utilized to identify patients at risk. With the widespread adoption of electronic health records (EHR), automated alerts incorporating vital signs and risk indicators for severe illness have become integral in clinical workflows. The early identification of septic patients at risk for poor outcomes supports timely interventions. Given that the EASIX score reflects endothelial injury, it may serve as a tool for identifying patients at an increased risk of developing severe sepsis and experiencing poorer outcomes. Additionally, the EASIX score is a straightforward tool that relies on three commonly available laboratory values (creatinine, lactate dehydrogenase (LDH), and platelet count), making it an easy and practical screening tool for identifying patients at risk of severe sepsis and adverse outcomes. Therefore, our hypothesis was that a higher EASIX score at the time of sepsis is associated with vasopressor support in critically ill PHO patients. Additionally, this exploratory study aimed to investigate the association of the EASIX score with survival in this high-risk population. A secondary goal was to compare the predictive performance of the EASIX score with the modified EASIX (mEASIX) score in this patient population.

## 2. Materials and Methods

### 2.1. Study Population

All children admitted to the intensive care unit (ICU) at St. Jude Children’s Research Hospital, a specialized pediatric hematologic–oncology hospital, with sepsis between July 2022 and December 2024 were included in the study. Patients were excluded if serum creatinine, LDH, or platelet lab data was unavailable within the first 24 h of ICU admission, or if a patient experienced multiple sepsis events during the same ICU stay—in which case only the first event was considered. If a patient experienced multiple sepsis events associated with separate ICU admissions, data from each of these events was included in the analysis.

### 2.2. Data Collection

The following ICU course data were collected: patient demographics, primary oncologic diagnosis, history of HCT, Pediatric Risk of Mortality (PRISM) 3 score, Pediatric Index of Mortality (PIM) 2, use of invasive mechanical ventilation (IMV) or positive pressure ventilation, use of vasopressor or inotropic infusion (including norepinephrine, epinephrine, dobutamine, phenylephrine, vasopressin, dopamine, and milrinone), renal replacement therapy (RRT), ICU length of stay, and survival. In addition, laboratory data, including serum creatinine, LDH, platelet, and C-reactive protein (CRP) within the first 24 h of ICU admission, were collected.

### 2.3. Definitions

The sepsis definition was based on improving pediatric sepsis outcomes (IPSO) collaborative definitions. IPSO, a collaborative, multicenter quality improvement network sponsored by the Children’s Hospital Association, focuses on improving sepsis outcomes by prompting early recognition and timely treatment. Patients with “IPSO sepsis” were identified either by (1) the presence of a standalone International Classification of Diseases, 10th edition (ICD − 10) code for severe sepsis/septic shock or (2) the receipt of the following sepsis treatments (blood culture obtained within 72 h and antibiotics delivered and either two fluid boluses administered OR bolus along with vasoactive agent given PLUS one of the following: ICU admission, lactate measurement, administration of vasoactive agent, use of infectious disease order set usage, or an ICD-10 code for sepsis assigned) [14].

EASIX score was calculated using the following formula: serum LDH level (U/L) × creatinine level (mg/dL)/platelet count (10^9^/L) [15]. Modified EASIX score was calculated based on the following formula: serum LDH level (U/L) × CRP (mg/dL)/platelet count (10^9^/L) [16].

### 2.4. Statistical Analysis

For the primary statistical analysis, EASIX and mEASIX were transformed and analyzed using log_2_. Two cut points of log2-EASIX, one for vasopressor classification and one for mortality classification, were derived to maximize the sum of the sensitivity and specificity (Youden method). Categorical variables were presented as frequencies with percentages, whereas median and interquartile range (IQR) were used to describe continuous variables. All continuous variables were without the assumption of normality and analyzed using the Kruskal–Wallis rank test (non-parametric *t* test). Categorical variables were compared using Fisher’s exact test. 

Odds ratios and the corresponding 95% confidence intervals were estimated using multivariable logistic regression models with continuous and dichotomized EASIX scores. Model adjustments were made for age, sex, and pre-sepsis HCT. Classification statistics were calculated from the classification table using a model-estimated probability threshold for assignment to positive outcome to 50%. 

Receiver operating characteristic (ROC) curves were used to assess the sensitivity, specificity, and area under the ROC curve (AUC) of the standard and modified EASIX score and each outcome (vasopressor use and mortality). 

## 3. Results

A total of 70 episodes of sepsis associated with ICU admission were identified. After applying the exclusion criteria, 53 patients with 65 sepsis events were included in the analysis (Supplemental Figure S1). The median age in our cohort was 14.9 [IQR 9.9 y] and the most common primary disease was hematologic malignancy (71%), and 48% received HCT (Table 1). In our cohort, 60% needed vasopressor support, 36% required IMV, and 22% underwent RRT. An infectious agent was identified in 69% of the cases, with bacterial infections comprising 53%, viral infections 14%, and fungal infections 2%. The survival to ICU discharge rate in our cohort was 79%.

### 3.1. EASIX Score and Outcome

The cut-off value of >2.5 was selected using the Youden method to compare the high log2-EASIX group versus the low log2-EASIX group. A high log2-EASIX was noted in 35% of the sepsis events (Table 1). The high log2-EASIX group had higher vasopressor requirements (87% versus 45% in the low log2-EASIX group, *p* = 0.001), higher rates of bacterial infection (74% versus 42%, *p* = 0.019), and an increased need for RRT (39% versus 12%, *p* = 0.024), along with a non-significant trend towards a greater use of IMV (52% versus 27%, *p* = 0.059). In addition, patients in the high log2-EASIX group experienced longer ICU stays (7 d versus 2 d in the low log2-EASIX group, *p* = 0.024) and an extended overall hospitalization (33 d versus 25 d, *p* = 0.029).

**Figure 1:**
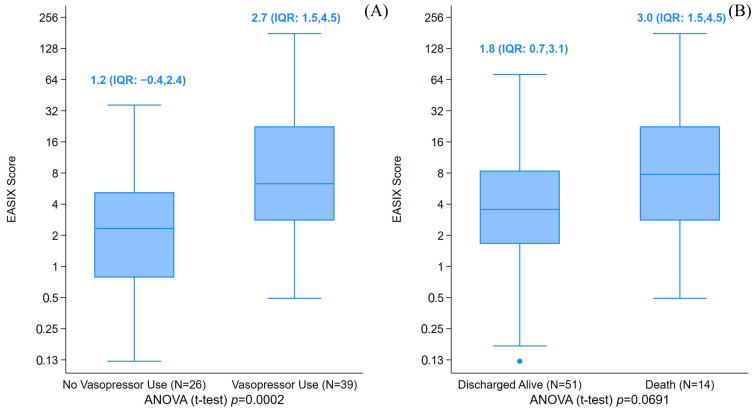
The box plot of the Endothelial Activation and Stress Index (EASIX) score by vasopressor use (panel (**A**)) and death probabilities (panel (**B**)). The boxplots show the summarized distribution and suspected outlying values for the EASIX score. (**A**) The median log2-EASIX score was 2.7 (IQR: 1.5–4.5) when vasopressors were used, versus 1.2 (IQR: −0.4,2.4) when not utilized. (**B**) The median log2-EASIX for patients that died in the ICU was 3.0 (IQR: 1.5–4.5), versus 1.8 (IQR: 0.7,3.1) when discharged alive. ^●^ represents outlying values.

When evaluating log2-EASIX as a continuous variable, children who required vasopressor support had a higher median value compared to those who did not (2.7 versus 1.2, *p* = 0.0002) (Figure 1).

**Figure 2:**
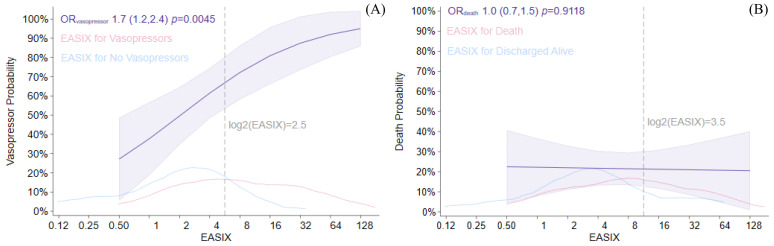
Endothelial Activation and Stress Index (EASIX) score associations with vasopressor use (panel (**A**)) and death probabilities (panel (**B**)). Vasopressor use and death probabilities are shown across the log2-EASIX distribution, with corresponding odds ratio estimates given with 95% confidence limits (purple). Individual kernel densities are provided for death and vasopressors (red) as well as vasopressor-free stays and surviving discharges (blue). Reference lines for each cut point are provided (gray, dashed). Logistic regression models adjusted for age, sex, and pre-sepsis HCT. (**A**) Vasopressor use model interpretation: each doubling of EASIX was associated with an OR of vasopressor use of 1.7, *p* = 0.004. (**B**) Death model interpretation: our data suggested EASIX was not associated with mortality (OR = 1.0, *p* = 0.912).

Multivariable analysis revealed that a higher log2-EASIX was associated with an increased likelihood of vasopressor use with an odds ratio (OR) of 1.7 [(95% CI 1.2–2.4) *p* = 0.004] (Figure 2).

Furthermore, a log2-EASIX cut-off value > 2.5 was associated with an OR of 6.9 for requiring vasopressor support [(95% CI 1.7–27.8) *p* = 0.007]. This threshold demonstrated a sensitivity of 77%, a specificity of 50%, and a positive predictive value of 70% (Table 2).

### 3.2. EASIX Score and Survival

**Figure 3:**
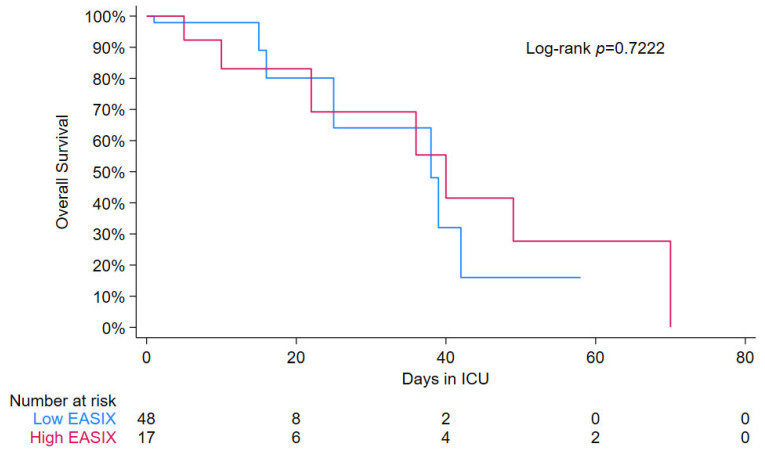
Kaplan–Meier curve for low vs. high EASIX (cut-point > 3.5). Kaplan–Meier curves comparing survival times between the low EASIX group (blue) and high EASIX group (red). The analysis shows a similar pattern between the groups, with a *p*-value of 0.7222 indicating the absence of statistical support above or below the cut point.

ICU survival rates did not differ significantly between the two groups (70% in the high log2-EASIX group versus 83% in the low log2-EASIX group, *p* = 0.221) (Table 1). The median log2-EASIX was 3 among children who did not survive ICU discharge, versus 1.8 in those who survived (*p* = 0.069). Overall survival was not statistically different for a high versus low log-2 EASIX (Figure 3). In addition, the multivariable analysis did not show an association between high log-2 EASIX and mortality (Figure 2).

### 3.3. EASIX Score Versus Modified EASIX Score

**Figure 4:**
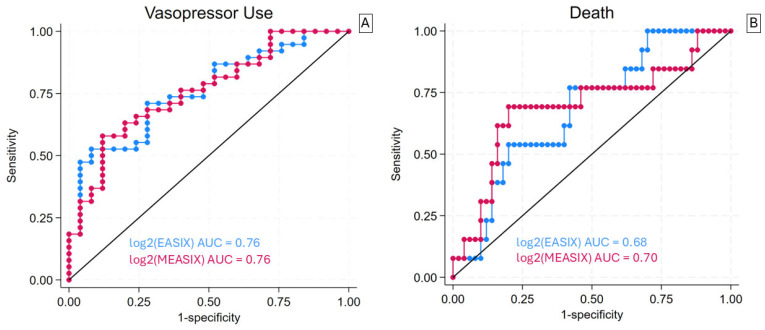
ROC curves for vasopressor use (panel (**A**)) and in-ICU mortality (panel (**B**)) of Endothelial Activation and Stress Index (EASIX) Score and a modified version that replaces measured serum creatinine with C-reactive protein. The receiver operating characteristic curve for all cut points of EASIX (blue) and MEASIX (red) for death and vasopressor use. The black diagonal line represents the cut point where, when above, the classifier makes predictions better than random guessing. (**A**) EASIX and MEASIX yielded a consistent area under the ROC curve (AUC) of 0.76 for vasopressor use. (**B**) EASIX and MEASIX yielded a similar AUC of 0.68 and 0.70, respectively, for in-ICU mortality.

Both the EASIX score and the mEASIX score showed a comparable performance in predicting the need for vasopressor support, each achieving an area under the curve (AUC) of 0.76 (Figure 4). Likewise, both scores demonstrated a similar AUC when predicting mortality (AUC ~0.69 for both).

## 4. Discussion

To the best of our knowledge, the present study is the first to investigate the association between EASIX and the need for vasopressor support in critically ill septic PHO patients. In our cohort, an elevated EASIX score was significantly associated with the need for vasopressor support. PHO patients with a log2-EASIX > 2.5 had a 6.9-fold increased likelihood of requiring vasopressors. Furthermore, a log2-EASIX > 2.5 was associated with additional adverse outcomes, including a higher RRT requirement, prolonged ICU stays, and an extended overall hospitalization. Previous studies have demonstrated that pre-transplant EASIX scores are associated with an increased mortality in patients undergoing HCT [17]. Similarly, in a cohort of 7504 adult ICU patients with sepsis, a higher log2-EASIX was associated with an increased risk of 28-day and 90-day mortality [11]. Although our study did not demonstrate a statistically significant association between EASIX scores and ICU survival, we observed a trend towards higher median log2 EASIX scores among non-survivors (3) compared with survivors (1.8). This trend suggests a possible association between higher EASIX scores and an increased mortality risk, which may not have reached statistical significance due to the limited sample size and study power. These findings underscore the need for larger, multicenter investigations to validate the prognostic utility of EASIX scores for mortality prediction in septic PHO patients.

Since the EASIX score reflects endothelial injury, it is plausible that higher scores predict severe sepsis phenotypes, characterized by the need for vasopressor support, RRT, and a prolonged ICU stay. This aligns with the central role the endothelium plays in sepsis pathogenesis. The endothelium is a dynamic organ involved in key homeostatic mechanisms including inflammation, coagulation, and vascular tone [18]. Endothelial cells express adhesion molecules that facilitate neutrophil transendothelial migration in response to cytokine signaling. They also actively secrete inflammatory cytokines and chemokines, amplifying the immune response. While this amplification can help fight infection, it can also be detrimental due to pathologic inflammation [18]. In sepsis, the normal mechanisms that preserve endothelial disruption are compromised, resulting in an increased vascular permeability and interstitial edema—key drivers of the organ dysfunction seen in septic patients [18]. Endothelium also contributes to the activation of the coagulation system during sepsis, shifting the balance toward a pro-coagulant state. This can manifest as disseminated intravascular coagulation, thrombotic thrombocytopenic purpura, or thrombocytopenia-associated multi-organ failure, all of which are linked to poor outcomes [10]. Additionally, the endothelium plays a crucial role in vascular tone regulation. Reactive oxygen species and reactive nitrogen species produced during sepsis can damage the endothelial glycocalyx, disrupting the endothelium’s ability to properly manage the balance between vasodilatation and vasoconstriction [10].

The modified EASIX score has been reported in some studies to be a predictor of cytokine release syndrome (CRS) and immune effector cell-associated neurotoxicity syndrome (ICANS) after CD19-directed chimeric antigen receptor (CD19-CAR) T-cell therapy [19,20,21]. The mEASIX score outperformed the EASIX score in these patients [20,21]. In our cohort, both EASIX and mEASIX demonstrated a comparable performance in predicting the need for vasopressor support. 

While several biomarkers of endothelial disruption have shown strong associations with poor outcomes in sepsis, their clinical utility at the bedside is limited, as they are not readily available [22]. In contrast, the EASIX score is derived from routinely obtained laboratory results, enabling its use at the bedside. This makes it a practical and accessible tool for identifying septic patients at increased risk of clinical deterioration and poor outcomes.

The limitations of our study include (1) its retrospective design and relatively small sample size, which may limit the detection of statistically significant associations; (2) the lack of a control group; and (3) the evaluation timeline, which was primarily confined to the first 24 h. Nonetheless, this is the first study to evaluate the predictive utility of the EASIX score in septic PHO patients. Complex pediatric HCT-IEC patients can have elevated EASIX and mEASIX scores from complications of therapy. Therefore, larger prospective studies are needed to confirm how these scores can be used in conjunction with other clinical information to identify patients at the highest risk for needing ICU interventions with sepsis.

In summary, the EASIX score was associated with a need for vasopressor support and RRT in septic PHO. If validated in a larger cohort, incorporating the EASIX score into sepsis screening protocols could aid in the early identification of high-risk patients who may benefit from closer monitoring and potentially help distinguish between distinct sepsis phenotypes.

## Figures and Tables

**Table 1:** Characteristics of PHO in the low log-2 EASIX group versus the high log-2 EASIX group.

	Low EASIX	High EASIX	Total	*p*-Value
N	42 (64.6%)	23 (35.4%)	65 (100.0%)	
Sex				
Female	17 (40.5%)	15 (65.2%)	32 (49.2%)	0.072
Male	25 (59.5%)	8 (34.8%)	33 (50.8%)	
Race				
White	27 (64.3%)	15 (65.2%)	42 (64.6%)	0.800
Black	9 (21.4%)	6 (26.1%)	15 (23.1%)	
Other	6 (14.3%)	2 (8.7%)	8 (12.3%)	
Diagnosis Group				
Hematologic Malignancy	31 (73.8%)	15 (65.2%)	46 (70.8%)	0.608
Benign Hematologic Disease	6 (14.3%)	3 (13.0%)	9 (13.8%)	
Solid Tumor	5 (11.9%)	5 (21.7%)	10 (15.4%)	
Age at ICU Admission (y)	14.5 [10.8]	15.1 [10.1]	14.9 [9.9]	0.641
HCT				
No	24 (57.1%)	10 (43.5%)	34 (52.3%)	0.313
Yes	18 (42.9%)	13 (56.5%)	31 (47.7%)	
Sepsis After ICU Admission				
No	36 (85.7%)	17 (73.9%)	53 (81.5%)	0.319
Yes	6 (14.3%)	6 (26.1%)	12 (18.5%)	
Vasopressor Use				
No	23 (54.8%)	3 (13.0%)	26 (40.0%)	0.001 *
Yes	19 (45.2%)	20 (87.0%)	39 (60.0%)	
Death				
No	35 (83.3%)	16 (69.6%)	51 (78.5%)	0.221
Yes	7 (16.7%)	7 (30.4%)	14 (21.5%)	
Any Infection				
No	16 (39.0%)	4 (17.4%)	20 (31.2%)	0.095
Yes	25 (61.0%)	19 (82.6%)	44 (68.8%)	
Bacterial Infection				
No	24 (58.5%)	6 (26.1%)	30 (46.9%)	0.019 *
Yes	17 (41.5%)	17 (73.9%)	34 (53.1%)	
Viral Infection				
No	33 (80.5%)	22 (95.7%)	55 (85.9%)	0.140
Yes	8 (19.5%)	1 (4.3%)	9 (14.1%)	
Fungal Infection				
No	41 (100.0%)	22 (95.7%)	63 (98.4%)	0.359
Yes	0 (0.0%)	1 (4.3%)	1 (1.6%)	
IMV				
No	30 (73.2%)	11 (47.8%)	41 (64.1%)	0.059
Yes	11 (26.8%)	12 (52.2%)	23 (35.9%)	
RRT				
No	37 (88.1%)	14 (60.9%)	51 (78.5%)	0.024 *
Yes	5 (11.9%)	9 (39.1%)	14 (21.5%)	
Lactated Ringer’s				
No	12 (28.6%)	6 (26.1%)	18 (27.7%)	1.000
Yes	30 (71.4%)	17 (73.9%)	47 (72.3%)	
ICU Length of Stay (d)	2.0 [10.0]	7.0 [20.0]	2.0 [15.0]	0.024 *
Hospital Length of Stay (d)	24.5 [36.0]	33.0 [37.0]	27.0 [37.5]	0.029 *
Platelet Count (10^3^/mm^3^)	79.0 [119.0]	17.0 [37.0]	45.0 [104.0]	<0.001 *
C-Reactive Protein (mg/dL)	7.7 [13.1]	16.3 [19.1]	11.2 [18.7]	0.022 *
LDH (U/L)	288.5 [310.0]	363.0 [1615.0]	314.0 [417.0]	0.063
Creatinine (mg/dL)	0.5 [0.4]	0.8 [0.9]	0.5 [0.4]	0.002 *
PIM2	−3.0 [0.4]	−3.0 [1.8]	−3.0 [1.5]	0.428
PRISM3	5.5 [7.5]	14.0 [14.0]	8.0 [11.0]	0.004 *

Values represented as median [IQR] for continuous data and frequency (%) for categorical data. Log2-EASIX Score > 2.5 characterizes the high EASIX group. * Statistical significance with *p* < 0.05; PHO, pediatric hematology–oncology; EASIX, Endothelial Activation and Stress Index; ICU, intensive care unit; HCT, hematopoietic cell transplant; IMV, invasive mechanical ventilation; RRT, renal replacement therapy; LDH, lactate dehydrogenase; PIM, Pediatric Index of Mortality; PRISM, Pediatric Risk of Mortality.

**Table 2:** The classification performance of each EASIX cut point.

	Vasopressor Use Log2-EASIX > 2.5	Death Log2-EASIX > 3.5
OR (95% CI) *p*-value	6.9 (1.7, 27.8) *p* = 0.007	2.1 (0.4, 10.7) *p* = 0.351
Sensitivity	77%	57%
Specificity	50%	94%
Positive Predictive Value	70%	73%
Negative Predictive Value	59%	89%
Correctly Classified	66%	86%

EASIX, Endothelial Activation and Stress Index; OR, odds ratio.

## Data Availability

The raw data supporting the conclusions of this article will be made available by the authors on request.

## Supplementary Materials

The following supporting information can be downloaded at https://www.mdpi.com/article/10.3390/jcm14197105/s1. Figure S1: Study flow diagram; Table S1: The classification performance of each risk score cut point.

## Abbreviations

The following abbreviations are used in this manuscript:

EASIX	Endothelial Activation and Stress Index
mEASIX	Modified Endothelial Activation and Stress Index
PHO	Pediatric hematology oncology
HCT	Hematopoietic cell transplant
RRT	Renal replacement therapy
ICU	Intensive care unit
LDH	Lactate dehydrogenase
OR	Odds ratio
AUC	Area under curve
ROC	Receiver operating characteristic
CRP	C-reactive protein
PIM	Pediatric Index of Mortality
PRISM	Pediatric Risk of Mortality
IMV	Invasive mechanical ventilation
ICD	International Classification of Diseases
CRS	Cytokine release syndrome
ICANS	Immune effector cell-associated neurotoxicity syndrome
CD19-CAR	CD19-directed chimeric antigen receptor
SOS	Sinusoidal obstruction syndrome
