# Supplementary material for: Unraveling the Easix Score: Its Association with Vasopressor Need in Critically Ill Septic Pediatric Hematology–Oncology Patients
Source: J Clin Med. 2025 Oct 9;14(19):7105. doi: 10.3390/jcm14197105 (PMC12524857; doi:10.3390/jcm14197105)
Supplement: Supplementary file 1 [file jcm-14-07105-s001.zip › jcm-3810718-supplementary.pdf]

Supplementary Materials

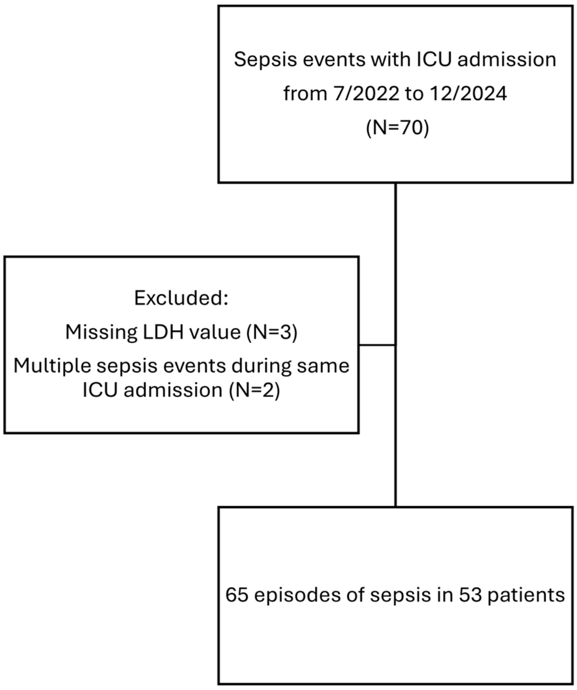

Supplemental Figure S1. Study flow diagram

To explore the comparison of the predictive performance of the EASIX score with other risk score biomarkers, we calculated the PRISM3 score. The log2-PRISM3 cut-off value > 3.6 was associated with an OR of 13.1 for requiring vasopressor support [(95% CI 2.3–75.1) p = 0.004]. This threshold demonstrated a sensitivity of 77%, a specificity of 68%, and a positive predictive value of 80% (Appendix Table A1). Although the performance was comparable with respect to in-ICU mortality, the association of PRISM3 with vasopressor use warrants additional exploration in a setting that limits missing data.

Table S1. The classification performance of each risk score cut point.

	Vasopressor Use		Death	
	**Log2-EASIX > 2.5**	**log2(PRISM3) > 3.6**	**Log2-EASIX > 3.5**	**log2(PRISM3) > 3.6**
**OR (95% CI) *p*-value**	6.9 (1.7, 27.8) p = 0.007	13.1 (2.3, 75.1) p = 0.0038	2.1 (0.4, 10.7) p = 0.351	2.4 (0.4, 15.4) p = 0.3457
**Sensitivity**	77%	77%	57%	55%
**Specificity**	50%	68%	94%	95%
**Positive Predictive Value**	70%	80%	73%	75%
**Negative Predictive Value**	59%	65%	89%	88%
**Correctly Classified**	66%	74%	86%	86%
**Data availability**	100% (65/65)	77% (50/65)	100% (65/65)	77% (50/65)
